# Homœopathic Therapeutics

**Published:** 1890-06

**Authors:** 


HOMŒOPATHIC THERAPEUTICS. Third rewritten and enlarged edition. By Samuel Lilienthal, M.D. Hahnemann Publishing House, F. E. Bœricke, 921 Arch Street, Philadelphia; pp. 1154; royal octavo, half morocco, $8.00; cloth, $7.00.

This, the third edition of Dr. Lilienthal's well-known book, represents half a century of the author's experience in the practice of medicine, to which is added all experience obtainable from other physicians.

The work is arranged alphabetically according to the names of the diseases. Under each of these names are given the most important remedies with their characteristic indications attached. The most completely verified of these indications are printed in bold-face type. All, however, are well authenticated as far as we could discover, the celebrated "key-notes" of Dr. H. N. Guernsey being included. After these indications follows a short and comprehensive repertory for that particular disease. Thus an additional help is afforded for the selection of the most similar remedy. We should think this work would be a most valuable aid to the earnest seeker after the simillimum.

